# Proliferation of the biocontrol agent *Fusarium oxysporum* f. sp. *strigae* and its impact on indigenous rhizosphere fungal communities in maize under different agro-ecologies

**DOI:** 10.1016/j.rhisph.2016.06.002

**Published:** 2016-06

**Authors:** Judith Zimmermann, Mary K. Musyoki, Georg Cadisch, Frank Rasche

**Affiliations:** Institute of Agricultural Sciences in the Tropics (Hans-Ruthenberg-Institute), University of Hohenheim, Stuttgart, Germany

**Keywords:** *Fusarium oxysporum* f.sp. *strigae*, 18S rDNA, Quantitative PCR, TRFLP analysis, maize

## Abstract

Our objectives were to (1) monitor the proliferation of the biocontrol agent (BCA) *Fusarium oxysporum* f. sp. *strigae* strain “Foxy-2”, an effective soil-borne BCA against the parasitic weed *Striga hermonthica*, in the rhizosphere of maize under different agro-ecologies, and (2) investigate its impact on indigenous rhizosphere fungal community abundance and composition. Field experiments were conducted in Busia and Homa Bay districts in western Kenya during two cropping seasons to account for effects of soil type, climate, growth stage and seasonality. Maize seeds were coated with or without “Foxy-2” and soils were artificially infested with *S. hermonthica* seeds. One treatment with nitrogen rich organic residues (*Tithonia diversifolia*) was established to compensate hypothesized resource competition between “Foxy-2” and the indigenous fungal community. Rhizosphere soil samples collected at three growth stages (i.e., EC30, EC60, EC90) of maize were subjected to abundance measurement of “Foxy-2” and total indigenous fungi using quantitative polymerase chain reaction (qPCR) analysis. Terminal restriction fragment length polymorphism (TRFLP) analysis was used to assess potential alterations in the fungal community composition in response to “Foxy-2” presence. “Foxy-2” proliferated stronger in the soils with a sandy clay texture (Busia) than in those with a loamy sand texture (Homa Bay) and revealed slightly higher abundance in the second season. “Foxy-2” had, however, only a transient suppressive effect on total indigenous fungal abundance which ceased in the second season and was further markedly compensated after addition of *T. diversifolia* residues. Likewise, community structure of the indigenous fungal community was mainly altered by maize growth stages, but not by “Foxy-2”. In conclusion, no adverse effects of “Foxy-2” inoculation on indigenous fungal rhizosphere communities were observed corroborating the safety of this BCA under the given agro-ecologies.

## 1 Introduction

*Striga hermonthica* (Del.) Benth. is an endemic parasitic weed of maize (*Zea mays* L.) and other cereal crops including sorghum (*Sorghum bicolor* L.), millet (*Pennisetum americanum* (L.) Leeke) and rice (*Oryza sativa* L.) which are main staple crops in Sub-Saharan Africa (Ejeta, 2007; Elzein and Kroschel, 2004; Marley et al., 2004). In western Kenya, *S. hermonthica* infests about 76% of total area under maize and sorghum causing annual crop loss deemed equivalent to 41 million US$ (Kanampiu et al., 2002, Vanlauwe et al., 2008).

The *Fusarium oxysporum* f. sp. *strigae* (Fos) strain “Foxy-2” was isolated from diseased *S. hermonthica* plants (Abbasher et al., 1995). This strain was proven to be effective in the suppression of all development stages of *S. hermonthica* ranging from germination to flowering (Elzein and Kroschel, 2004, Ndambi et al., 2011). Field experiments in Burkina Faso, Benin and Nigeria confirmed the combination of “Foxy-2” along with *Striga*-tolerant crop varieties as an effective integrated control approach against *S. hermonthica* (Schaub et al., 2006, Venne et al., 2009). Avedi et al. (2014) and Venne et al. (2009) showed, however, lack in control consistency of the biocontrol approach under field conditions, presumably as a consequence of differing environmental conditions across agro-ecological zones with different soil types, as well as rainfall and temperature patterns (Gerbore et al., 2013, Velivelli et al., 2014). Thus, a thorough understanding of environmental conditions which promote the proliferation and persistence of the BCA “Foxy-2” is required to ensure consistent and sustained *S. hermonthica* control. In this context, the monitoring of “Foxy-2” proliferation under contrasting soil conditions was issued by Zimmermann et al. (2015) who, using a Fos specific and quantitative monitoring tool, confirmed under controlled conditions that “Foxy-2” proliferation was determined by soil texture and promoted by the amendment of nitrogen (N)-rich organic resources. The latter fact requires particular attention since “Foxy-2” is a soil borne fungus and proliferates saprophytically and endophytically in crop rhizospheres and roots, respectively, where it has to compete with indigenous microorganisms for organic resources (Ndambi et al., 2011).

Potential competition between “Foxy-2” and indigenous microorganisms can determine the proliferation of the BCA and, in turn, may alter the abundance and composition of indigenous microbial communities. Soil microorganisms maintain important soil functions including nutrient cycling, suppression of soil-borne plant pathogens as well as promotion of plant growth (Compant et al., 2005, Liu et al., 2007, van der Heijden et al., 2008). Hence, it needs to be confirmed that the release and successful proliferation of “Foxy-2” in soils does not alter negatively the abundance and community composition of functionally relevant indigenous soil microorganisms. The exclusion of adverse effects of “Foxy-2” on non-target organisms is mandatory for the official registration of the BCA by regulatory authorities of Sub-Saharan African countries which generally oblige, according to international registration regulations, a profound risk assessment of BCAs (FAO, 2006, OECD, 2014). In this context, recent studies by Musyoki et al. (2015) and Zimmermann et al. (in press in “Fungal Ecology”) emphasized that the BCA “Foxy-2” exposed no negative effects on the abundance and community composition of indigenous soil prokaryotic and fungal populations. However, as these studies were conducted under short-term and controlled laboratory incubation conditions, progressive field studies are required over extended time periods considering additional factors such as seasonality and crop growth stages that determine the dynamics of rhizosphere microbial communities (Hai et al., 2009, Rasche et al., 2006, Marschner et al., 2003, Rasche et al., 2014).

We hypothesized that “Foxy-2” proliferation in soils is strongly controlled by agro-ecological conditions including soil type and climate, as well as organic residue inputs, seasonality and crop growth stage. Secondly, it was hypothesized that “Foxy-2” presence induces a considerable resource competition for indigenous fungi in the maize rhizosphere inducing alterations of their abundance and community composition. To account for this, we further hypothesized that this resource competition could be compensated by the application of N-rich organic residues (e.g., *T. diversifolia,* Chivenge et al., 2009). To test these hypotheses, the presented research considered two main objectives: (1) Monitor the proliferation of the BCA “Foxy-2” and (2) investigate potential alterations in total indigenous fungal abundance and composition due to “Foxy-2” exposure under field conditions in western Kenya. Both objectives were assayed at three distinct growth stages of maize cultivated in two contrasting field sites during two cropping seasons.

## 2 Material and methods

### 2.1 Fungal biocontrol agent

The Fos isolate “Foxy-2” was obtained from *S. hermonthica* collected from North Ghana (Abbasher et al., 1995). Taxonomic identification of the isolate was confirmed by the Julius-Kühn-Institut (JKI), Berlin, Germany, where it is deposited under accession number “BBA-67547-Ghana”. Since then, the isolate is preserved at −80 °C at the Institute of Agricultural Sciences in the Tropics, University of Hohenheim, Stuttgart, Germany.

### 2.2 Field experiments

#### 2.2.1 Study site description

The field experiments were carried out in post-entry quarantine facilities at the Agricultural Training Centre field stations in Western Kenya. Two study sites (Busia, 0° 26'S–34° 15′ E; 1200 m above sea level (a.s.l.); Homa Bay, 0° 40′–0°S and 0° 34° 50′E; 1305 m a.s.l.) were chosen because of the reported high *S. hermonthica* infestation in these areas (de Groote et al., 2008). The study areas have bimodal rainfall patterns with two growing seasons, the first rainy season with long rains (LR) from March to August and second rainy season with short rains (SR) from September to January. Busia district received 1157 mm precipitation in the SR of 2012/2013 and 606 mm in the LR of 2013, while the mean temperature was 27.4 °C and 26.8 °C in the SR and LR of 2012/2013, respectively. Homa Bay district received 383 mm precipitation in SR of 2012/2013 and 481 mm in the LR of 2013, while the mean temperature was 29.5 °C and 29.0 °C in the SR and LR of 2012/2013, respectively. The soils at Homa Bay were classified as vertic Phaeozems with a loamy clay texture (49% clay, 19% silt, 32% sand), while Busia has orthic Acrisols with a sandy clay texture (33% clay, 22% silt, 45% sand) (IUSS Working Group WRB, 2015).

#### 2.2.2 Field experiment setup and rhizosphere sampling

The study covered two seasons (SR; September 2012 to January 2013; LR; April 2013 to August 2013). The sites were left fallow for a year before the experiment was established. The fallow in Busia consisted of short grasses (e.g., *Digitaria scalarum*), while the fallow at Homabay consisted of grasses (*Digitaria scalarum*), and weeds such as black nightshade (*Solanum nigrum*) and thorn apples (*Datura stramonium*). *Zea mays* L. variety ‘WH507’ (provided by Western Seed Company Ltd., Kitale, Kenya), which is tolerant to *S. hermonthica* and of high preference by smallholder farmers in western Kenya, was planted in 3 m×2.7 m plots with a row spacing of 70 cm×30 cm. The experiment was laid out in a randomized complete block design (RCBD) with three replicates and comprised of three treatments: (i) uncoated maize and *S. hermonthica* (C+S), (ii) coated maize with “Foxy-2” and *S. hermonthica* (F+S), and (iii) coated maize with “Foxy-2”, *S. hermonthica* and *Tithonia diversifolia* residues (F+S+T). This experimental layout was repeated in the second season.

Land was prepared by hand digging and two maize seeds per hill were planted at a depth of approximately 3 cm. One tablespoonful of a *S. hermonthica* seed–sand mixture (1:4 with approximately 1000 *S. hermonthica* seeds) was placed in every planting hole (Avedi et al., 2014). All treatments received diammonium phosphate (23.5 kg N ha^−1^, 60 kg P_2_O_5_ ha^−1^) at sowing. For treatments C+S and F+S, additional N was split applied in the form of calcium ammonium nitrate (CaNH_4_NO_3_) at a rate of 120 kg N ha^−1^ with 1/3 and 2/3 added 3 and 8 weeks after sowing, respectively. For treatment F+S+T, N was applied as fresh *T. diversifolia* leaf and stem material (5 t dry weight ha^−1^ to supply similar levels to 120 kg of inorganic N (Gacheru and Rao, 2001)). The organic residue was hand-incorporated to a soil depth of 0–15 cm at the onset of each rainy season. Two weeks after germination, seedlings were thinned to 1 plant per hole. Hand weeding was done every 2 weeks for all weeds except *S. hermonthica*.

Rhizosphere samples (approximately 50 g) were collected according to standard procedures (Milling et al., 2005) at EC30 (early leaf development stage, Zadoks et al., 1974), EC60 (flowering stage), and EC90 (senescence stage) by shaking the roots of three plants per plot to remove non-rhizosphere soil. Rhizophere soil samples were then mixed to one composite sample. Soils were freeze-dried to avoid further microbial activity and stored in a dark and dry place. One proportion of the obtained rhizosphere soil samples was used to study the impact of “Foxy-2” on indigenous prokaryotic communities and for soil chemical analysis (Musyoki et al., 2016) while the other proportion was used in the present study to assess the “Foxy-2” abundance and its impact on indigenous fungal communities.

### 2.3 Analysis of fungal communities

#### 2.3.1 DNA extraction from rhizosphere samples

Total genomic DNA from rhizosphere samples was extracted using the Fast DNA® Spin Kit for Soil (MP Biomedicals, Solon, OH, USA) following the manufacturer's instructions with slight modifications. Briefly, 0.4 g freeze-dried soil was bead-beated for 30 s with a beating power of 5.5 m s^−1^ using a FastPrep®-24 Instrument (MP Biomedicals). Concentration and quality of DNA were determined on a Nanodrop ND-1000 (Nanodrop Technologies, Wilmington, DE, USA) and DNA was stored at −20 °C.

A soil spiking experiment was conducted including the two soils of the field experiments (i.e., Busia and Homa Bay) to account for soil type depending DNA extraction efficiencies influencing fungal gene copy recovery. Briefly, 400 mg of freeze dried soil samples obtained from control sets of the field experiment were transferred into the beat beating tubes of the DNA extraction kit (MP Biomedicals). Soil samples in tubes were spiked with cloned “Foxy-2” amplicons of known concentration (10^3^ “Foxy-2” gene copies). Recovery of “Foxy-2” amplicons after DNA extraction was determined using the qPCR protocol with “Foxy-2” specific oligonucleotides Kb1:Kb2 as described in Section 2.3.2. Results of the soil spiking experiment verified that DNA extraction efficiency was soil type independent.

#### 2.3.2 “Foxy-2” abundance

Quantification of “Foxy-2” gene copy numbers in soils was performed using Fos-specific oligonucleotides Kb1 (5′-GGACGAACTGACAGCCCTAC-3′) and Kb2 (5′-GTAACCGTAATATTGTTCAGAGCTC-3′) (Zimmermann et al., 2015). Each reaction (20 μl) contained 10 ng rhizosphere soil DNA template, 10 μl of Power SYBR® Green PCR Master Mix (Applied Biosystems, Foster City, CA, USA), 0.2 µM of each oligonucleotide Kb1 and Kb2, as well as 0.2 μl T4 gene 32 protein (500 ng μl^−1^, MP Biomedicals). A cloned amplicon was used as standard in 10-fold serial dilutions of known DNA concentration. PCR runs were performed on a StepOnePlus™ Real-Time PCR System (Applied Biosystems). Reactions started with initial denaturation at 95 °C for 10 min, followed by 45 cycles of denaturation at 94 °C for 30 s, annealing at 62 °C for 30 s, and polymerization at 72 °C for 1 min as well as one additional step at 77 °C for 30 s for signal detection. Occasionally, small peaks occurred in the melting curve between 72 and 76 °C due to primer dimers not detected by electrophoresis in a 1.5% agarose gel (data not shown). To avoid measurement of fluorescence signal emitted by these primer dimers, fluorescence of target amplicons (melting temperature (Tm)=81.8 °C) was detected at 77 °C. Each DNA sample was processed in triplicate reactions, while standards were run in duplicates. Melting curve analysis of amplicons was conducted to confirm that fluorescence signals originated from specific amplicons and not from primer dimers or other artefacts. An average reaction efficiency of 96.8% was achieved. Quantification of gene copies was calculated by comparing values of threshold cycles (Ct) to values of crossing points of the linear regression line of the standard curve using StepOne™ software version 2.2 (Applied Biosystems).

#### 2.3.3 Total fungal abundance

Total fungal abundance was assessed using oligonucleotides targeting the gene coding for a part of the small ribosomal subunit (18 S). Quantification of 18S rDNA gene copy numbers in soils was performed using oligonucleotides FF390 (5′-CGATAACGAACGAGACCT-3′) and FR1 (5′-AICCATTCAATCGGTAITCATTCA-3′) (Vainio and Hantula, 2000) and a cloned amplicon as standard (Kamolmanit et al., 2013). Each reaction (20 μl) contained 5 ng DNA template, 10 μl of Power SYBR® Green Master Mix (Applied Biosystems), 0.2 μl T4 gene 32 protein (500 ng μl^−1^, MP Biomedicals), and 0.4 μM of each oligonucleotide. Cycling started with initial denaturation at 95 °C for 10 min, followed by 45 cycles of denaturation at 94 °C for 30 s, annealing at 50 °C for 30 s and polymerization at 70 °C for 1 min. Average reaction efficiency was 92.5% and quantification of gene copies was done as described above.

It needs to be considered that the inoculated Fos strain “Foxy-2” is part of the total fungal abundance. Hence, it was likely that the abundance of “Foxy-2” was superimposed on the abundance of the indigenous fungal population. To account for this, “Foxy-2” abundance was subtracted from total fungal abundance as described in the following procedure. “Foxy-2” was propagated in 5 ml potato dextrose broth at 28 °C for 3 days, followed by DNA extraction (UltraClean Microbial DNA Isolation Kit, MO BIO Laboratories Inc., Carlsbad, CA). Concentration and quality of “Foxy-2” DNA were determined as described above. Five ng of “Foxy-2” DNA was used as template #1 for Fos-specific qPCR (using oligonucleotides Kb1:Kb2 with the protocol published in Zimmermann et al. (2015)) and template #2 for 18S rDNA qPCR (see above). The 5 ng “Foxy-2” DNA template used for both qPCR assays corresponded to 2.3*10^5^ “Foxy-2” gene copies and 4.6*10^5^ 18S rDNA gene copies resulting in a ratio of 1:2 between “Foxy-2” and 18S rDNA gene copies. Accordingly, the previously measured “Foxy-2” gene copy numbers in the soils from Busia and Homa Bay were first multiplied with factor 2 and then subtracted from total 18S rDNA gene copy numbers. This calculation resulted in the adjusted 18S rDNA gene copy numbers reflecting the abundance of the total indigenous fungal population.

#### 2.3.4 Total fungal community composition

The total fungal community composition was studied by terminal restriction fragment length polymorphism (TRFLP) analysis using the same oligonucleotide set as applied for 18S rDNA qPCR (Vainio and Hantula, 2000, Kamolmanit et al., 2013). T-RFLP analysis was only performed on rhizosphere soil samples obtained during the second study season (LR). The 18S rDNA gene was amplified in 25-μl reactions containing 5 ng DNA template, 1× PCR buffer, 2 U Taq DNA polymerase (Bioline GmbH, Luckenwalde, Germany), 0.2 mM of each deoxynucleoside triphosphate, 0.4 μM of each oligonucleotide (FF390:FR1), and 1 mM MgCl_2_. The forward oligonucleotide FF390 was labelled with the fluorescent dye FAM-6. PCRs were started with initial denaturation at 95 °C for 1 min, followed by 30 cycles consisting of a denaturation at 95 °C for 30 s, an annealing step at 52 °C for 45 s, and elongation at 72 °C for 2 min. Reactions were completed with a final elongation step at 72 °C for 10 min. Amplicons were purified using the Invisorb Fragment CleanUp Kit (Stratec Biomedical AG, Birkenfeld, Germany) following the manufacturer's instructions. For digestion, 200 ng of amplicons were incubated with 5 U *Msp*I restriction endonuclease (Promega GmbH, Mannheim, Germany) at 37 °C for 4 h followed by 65 °C for 20 min enzyme inactivation. Digested products were desalted with Sephadex™ G-50 (GE Healthcare) (Rasche et al., 2006) and amended with 7.75 μl Hi-Di formamide (Applied Biosystems) and 0.25 μl internal size standard GeneScan™−500 ROX™ (Applied Biosystems). Mixtures were denaturated at 95 °C for 2 min, followed by immediate chilling on ice. TRFLP profiles were recorded on an ABI Genetic Analyzer 3130 (Applied Biosystems). Peak Scanner software (version 1.0, Applied Biosystems) was used to compare relative lengths of terminal-restriction fragments (T-RFs) with the internal size standard and to compile electropherograms into numeric data sets, in which T-RF length and height >100 fluorescence units (Fredriksson et al., 2014) were used for statistical profile comparison. TRFLP profiles used for statistical analyses were normalized according to Dunbar et al. (2000).

A requirement for analysing “Foxy-2” induced alterations in indigenous fungal community composition was the deletion of “Foxy-2” T-RF from TRFLP profiles. The explicit “Foxy-2” T-RF was deleted from fungal TRFLP profiles using the following procedure: “Foxy-2” was propagated in 5 ml potato dextrose broth at 28 °C for 3 days. DNA was extracted (UltraClean Microbial DNA Isolation Kit, MO BIO Laboratories Inc., Carlsbad, CA), and quantified as described above. Five ng DNA was amplified in triplicate reactions using oligonucleotides FF390:FR1. Amplicons were purified (Invisorb^®^ Fragment CleanUp kit (Stratec Molecular GmbH)), quantified and sequenced with oligonucleotide FR1 (LGC Genomics GmbH, Berlin, Germany). 18S rDNA sequences of “Foxy-2” were submitted to http://www.restrictionmapper.org/ to identify the restriction cutting site with the enzyme Msp*I* used for TRFLP. The resulting T-RF of “Foxy-2” with 168 base pair length was deleted from all TRFLP profiles.

### 2.4 Statistical analysis

**Fig. 1:**
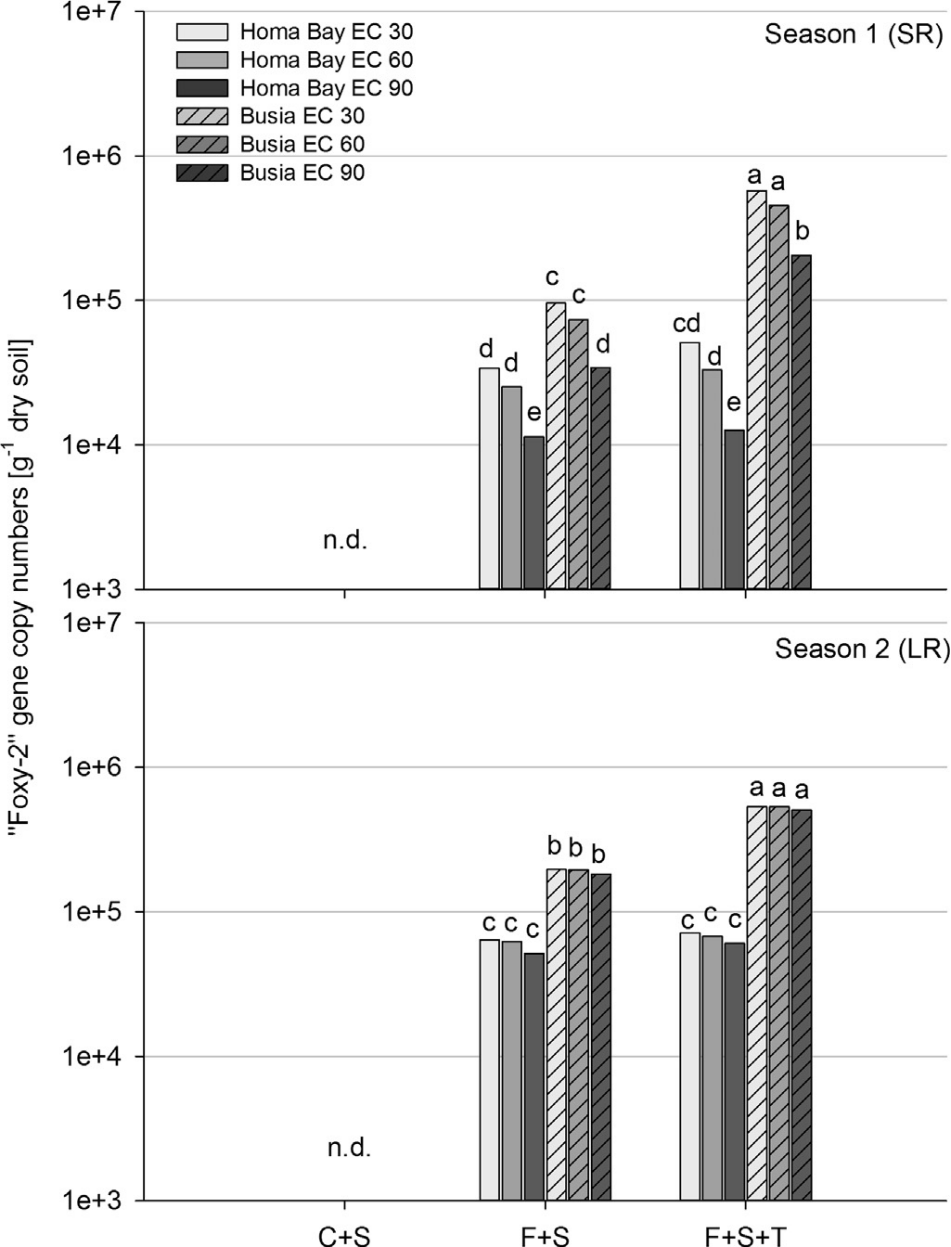
“Foxy-2” abundance (gene copy numbers obtained from qPCR analysis) during three maize growth stages (EC 30 (early leaf development stage), EC 60 (flowering stage), EC 90 (senescence stage)) in Season 1 (SR, short rains) and Season 2 (LR, long rains) at the two field sites Homa Bay and Busia. Treatments: uncoated maize and *S. hermonthica* (C+S), coated maize with “Foxy-2” and *S. hermonthica* (F+S), coated maize with “Foxy-2”, *S. hermonthica* and *Tithonia diversofolia* residues (F+S+T). Different letters indicate significant differences at *P*<0.05. Abbreviation n.d.=not detected.

**Fig. 2:**
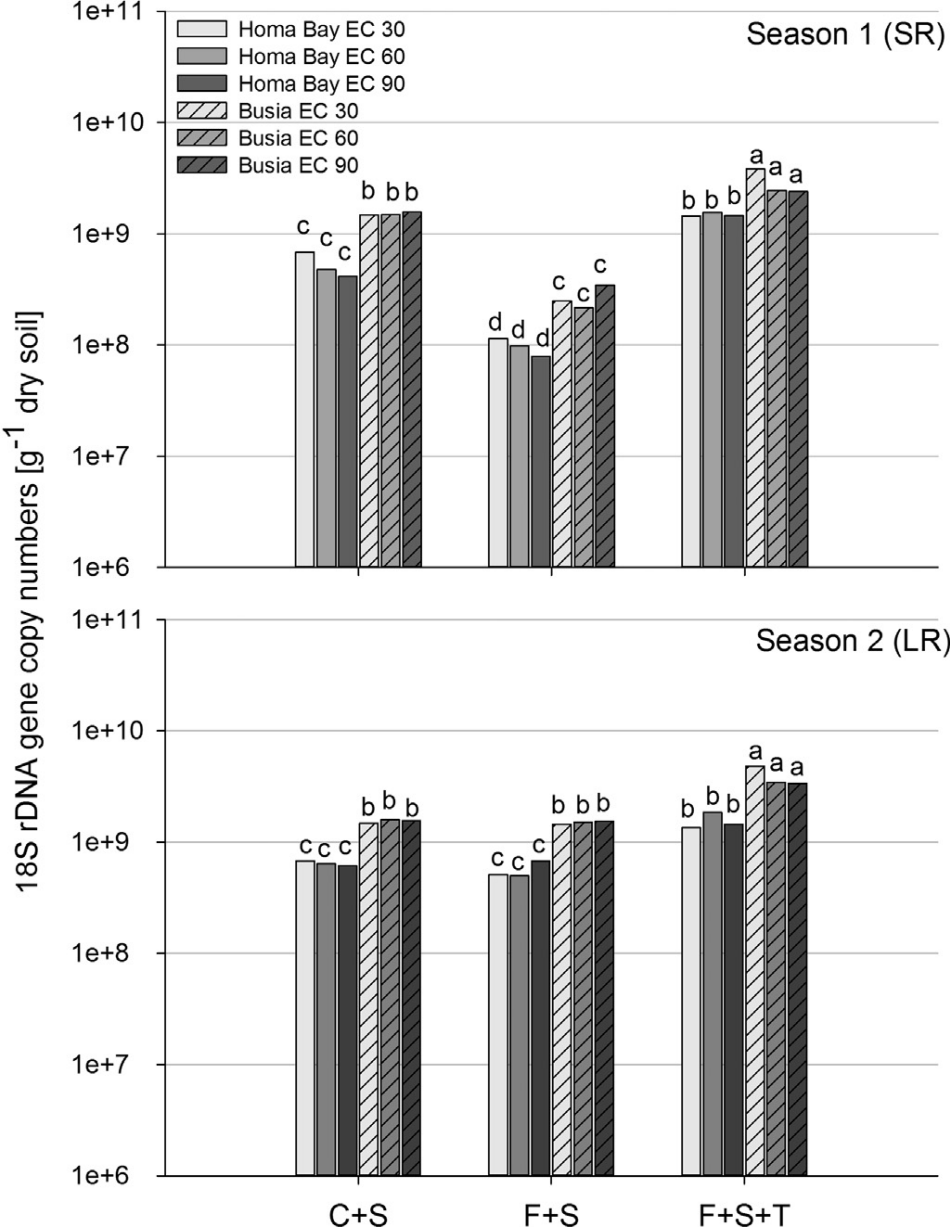
Total fungal abundance (18S rDNA gene copy numbers obtained from qPCR analysis) during three maize growth stages (EC 30 (early leaf development stage), EC 60 (flowering stage), EC 90 (senescence stage)) in Season 1 (SR, short rains) and Season 2 (LR, long rains) at the two field sites Homa Bay and Busia. Treatments: uncoated maize and *S. hermonthica* (C+S), coated maize with “Foxy-2” and *S. hermonthica* (F+S), coated maize with “Foxy-2”, *S. hermonthica* and *Tithonia diversofolia* residues (F+S+T). Different letters indicate significant differences at *P*<0.05.

Statistical analyses on obtained qPCR data sets (“Foxy-2” and 18S rDNA gene copy numbers) were performed using R software (Software R 3.0.1, R foundation for Statistical Computing, Vienna, Austria, http://www.R-project.org). QPCR data was log transformed to meet the assumptions of parametric statistical tests. Effects of factors “Treatment” (Control, “Foxy-2”, *T. diversifolia*), “Maize growth stage”, “Season” and “Field site” on abundance of both studied genes in qPCR were assessed using linear mixed-effects models in R with the “nlme” package (Pinheiro et al., 2016). Since repeated measures (three maize growth stages) were taken within each season, a random effect was used in the model to account for serial autocorrelation at each plot. Furthermore, the factor “Maize growth stage” was nested in “Season”. An autoregressive variance-covariance structure was fitted to compensate for the proximity of the observations. Least squares means comparison between factors was done using the Tukey´s range test (*P*<0.05). Bar charts displaying gene copy numbers (Fig. 1, Fig. 2) were shown without standard errors due to back transformation of the least square means. For statistical purpose, soil chemical data were retrieved from a parallel study by Musyoki et al. (2016), where analytical procedures were described. Soil chemical properties were obtained from each rhizosphere sample. The impact of soil chemical properties (Musyoki et al., 2016) on “Foxy-2” and total fungal abundance was assessed by adding the soil chemical parameters to the above described linear mixed-effects model as co-variables. The resulting model was stepwise reduced based on the Akaike information criterion (AIC) by using the “stepAIC” function of the R package “MASS” (Venables and Ripley, 2002).

TRFLP data sets generated for 18S rDNA were assayed based on Bray-Curtis similarity coefficients (Rees et al., 2005, Kamolmanit et al., 2013). The similarity matrix was used for analysis of similarity (ANOSIM) to test the hypothesis that total fungal composition was altered by factors “Treatment”, “Field site”, and “Maize growth stage”. ANOSIM is based on rank similarities between the sample matrix and produces a test statistic ‘*R*’ (Rees et al., 2005). A ‘global’ *R* was first calculated in ANOSIM, which evaluated the overall effect of a factor in the data set. This step was followed by a pair wise comparison, whereby the magnitude of *R* indicated the degree of separation between two tested communities. An *R* score of 1 indicated a complete separation, while 0 indicated no separation (Rees et al., 2005). Treatment separation was visualized by canonical analysis of principal coordinates (CAP) on the basis of Bray-Curtis similarity indices (Anderson and Willis, 2003). Calculation of similarity coefficients, ANOSIM and CAP were carried out using Primer for Windows version 6 (Primer-E Ltd., Plymouth, UK). To verify if considered soil chemical properties (Musyoki et al., 2016) were decisive for the observed treatment-driven community composition shifts of the total fungal population, the DistLM procedure of PERMANOVA+ in Primer v6 (Primer-E Ltd.) was used (Clarke and Gorley, 2006). This procedure calculates a linear regression between the diversity of fungal communities using the Shannon diversity index and log transformed soil chemical data (Legendre and Anderson, 1999).

## 3 Results

### 3.1 “Foxy-2” abundance

No “Foxy-2” was detected in the control treatments (C+S) which were consequently excluded from statistical analysis. Abundance of “Foxy-2” was higher in soils with a sandy clay texture (Busia) than in those with a loamy clay texture (Homa Bay) (*P*<0.001) (Fig. 1). A significantly higher abundance of “Foxy-2” was observed in the second season (*P*<0.05) at both field sites. *T. diversifolia* amendment stimulated “Foxy-2” abundance at Busia (*P*<0.001) but not at Homa Bay (*P*>0.05). A significant decrease in “Foxy-2” gene copies was detected from the first two maize growth stages (EC 30 and EC 60) to senescence (EC 90) in season 1 at both field sites (*P*<0.01). The impact of fixed factors (“Field site”, “Season”, “Maize growth stage” and “Treatment”) and their interactions on gene abundance (i.e., “Foxy-2”, total fungi) and soil chemical properties is summarized in Table 1, while the impact of soil chemical properties on gene abundance was assessed across field sites and within each field site (Table 2). Predicted values of “Foxy-2” gene copy numbers derived from the linear mixed effect model described in section 2.7 were significantly negative for pH (*P*<0.01, Table 2) and ammonia (*P*<0.05, Table 2) across field sites. In Busia, predicted values were negative for pH (*P*>0.01, Table 2) and positive for EON (*P*<0.05, Table 3). In Homa Bay, predicted “Foxy-2” gene copy numbers were negative for pH (*P*<0.01, Table 2), TC (*P*<0.05, Table 2) and EOC (*P*<0.05, Table 2).

### 3.2 Total fungal abundance

Total fungal abundance was higher in Busia compared to Homa Bay throughout all treatments (*P*<0.001, Fig. 2). “Foxy-2” inoculation induced a decrease in total fungal abundance only in season 1 at both field sites (*P*<0.001), while *T. diversifolia* amendment induced an increase in total fungal abundance at both field sites throughout both seasons (*P*<0.001). Maize growth stage revealed no effect on total fungal abundance (*P*>0.05). For the total fungal community, predicted gene copy numbers across field sites were negative for pH (*P*<0.01, Table 2) and TC (*P*<0.05, Table 2). In Busia, predicted values were negative for pH (*P*<0.01, Table 2) and positive for N_t_ (*P*<0.05, Table 2) and EOC (*P*<0.05, Table 2). In Homa Bay, predicted total fungal gene copy numbers were negative for TC (*P*<0.05, Table 2) and pH (*P*<0.05, Table 2).

### 3.3 Total fungal community composition

**Fig. 3:**
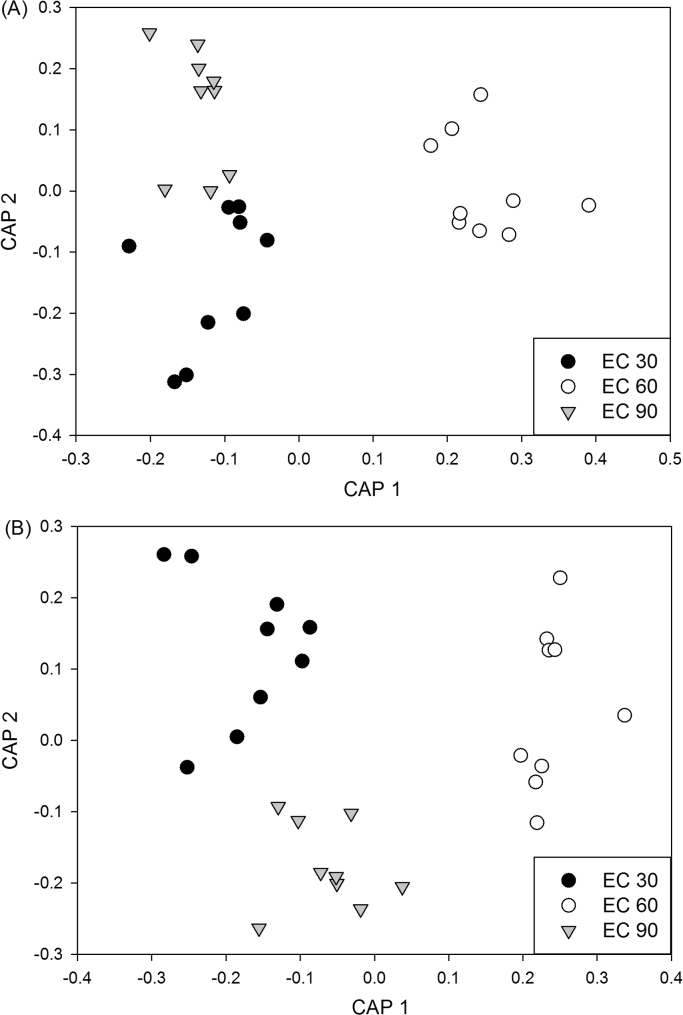
Canonical analysis of principal coordinates (CAP) ordination on the basis of Bray-Curtis similarity indices of normalized TRFLP data obtained from Msp*I*-digested 18S rDNA amplicons to visualize the differences in fungal community composition in Homa Bay (A) and Busia (B) according to the three maize growth stages (EC 30 (early leaf development stage), EC 60 (flowering stage), EC 90 (senescence stage)).

Analysis of similarity (ANOSIM) of TRFLP profiles revealed distinct total fungal community compositions between Busia and Homa Bay (*R*=1, *P*<0.001). Within each field site, factor “Treatment” showed no significant impact on fungal community composition (Homa Bay: global *R*=0.119, *P*>0.05; Busia: global *R*=0.073, *P*>0.05), while “Maize growth stage” induced strong alterations in fungal composition (Homa Bay: global *R*=0.410, *P*<0.01; Busia: global *R*=0.627, *P*<0.01) (Table 3, Fig. 3). At Homa Bay, EC 60 versus EC 90 resulted in the strongest fungal community alteration (*R*=0.580, *P*<0.01), while at Busia, fungal community separation was most pronounced between EC 30 and EC 90 (*R*=0.840, *P*<0.01). Shannon diversity indexes calculated from TRFLP data and log transformed soil chemical data revealed no significant correlations at both field sites (results not shown).

## 4 Discussion

Identification of favoured agro-ecological conditions for good establishment of the fungal BCA “Foxy-2” in the rhizosphere of crops is inevitable to optimally control the weed *S. hermonthica*. It was recently suggested that “Foxy-2” abundance is generally determined by physico-chemical soil properties and availability of organic N resources (Zimmermann et al., 2015), but effects of other relevant impact factors like crop growth stage and seasonality under natural field conditions are yet to be understood. It was our primary objective to address these questions through monitoring the proliferation of “Foxy-2” in the rhizosphere of crops cultivated at two agro-ecologically contrasting field sites in western Kenya considering three plant growth stages throughout two cropping seasons. Secondly, we investigated the potential impact of “Foxy-2” on indigenous rhizosphere fungal community abundance and composition considering N-rich organic residues to compensate for any resource competition.

### 4.1 “Foxy-2” prefers sandier soil textures with low pH and carbon background

Our study identified agro-ecological distinctions as substantial drivers of “Foxy-2” proliferation in assayed maize rhizospheres which was mainly attributed to contrasting soil textures, soil carbon background and pH, but also different climatic conditions (i.e., rainfall, temperature). “Foxy-2” exhibited a major preference towards the sandier soil type (i.e., Busia; orthic Acrisol) with low total carbon (TC) content and lower pH (~5) (Musyoki et al., 2016) which corroborated recent observations on “Foxy-2” by Zimmermann et al. (2015) and *Fusarium* spp. in general (Fang et al., 2012, Höper et al., 1995, Senechkin et al., 2014). Higher precipitation rates and lower mean temperatures at the field site Busia reinforced the influence of soil type on “Foxy-2” proliferation as was also revealed by Venne et al. (2009) showing increased “Foxy-2” efficacy in areas with high rainfall amounts.

We found clear indications that the abundance of “Foxy-2” was limited by the higher level of suppressiveness towards *Fusarium* spp. in the clayey soil (Homa Bay) with its strong soil organic carbon (SOC) background and higher pH (~7) (Musyoki et al., 2016), as indicated by predicted negative “Foxy-2” gene copy numbers in response to the respective soil parameters (Table 2). This finding is supported by earlier studies correlating soil suppressiveness to *Fusarium* spp. to abiotic soil characteristics such as clay content and pH (Höper et al., 1995, Yergeau et al., 2010). Furthermore, certain prokaryotes (i.e., *Bacillus* spp., *Pseudomonas* spp.) are acknowledged to act as antagonists of *Fusarium* spp. (Farooq and Bano, 2013, Köhl et al., 2015), thereby substantially contributing to soil suppressiveness. In this context, we found in a parallel study substantially higher abundance of archaea in the rhizosphere of maize grown on a clayey soil (Homa Bay) compared to a sandy soil (Busia) (Musyoki et al., 2016). A promoting effect of high soil pH (~7) and strong SOC background on archaea abundance was earlier acknowledged (Bengtson et al., 2012, Pereira e Silva et al., 2012). Moreover, our assumption on biotic factors (i.e., archaeal community) determining proliferation of “Foxy-2” is further supported by our finding that advanced maize growth development hampered “Foxy-2” abundance while stimulating archaea abundance in maize rhizospheres (Musyoki et al., 2016). Moreover, advances maize growth stages are acknowledged to stimulate mycorrhiza abundance (Grigera et al., 2007), a microbial group known to act as antagonists of *Fusarium* spp. (Hu et al., 2010, Shukla et al., 2015).

Interestingly, re-inoculation of “Foxy-2” in the second season resulted not only in higher abundance levels, but also in increased resilience of the BCA towards biotic factors (i.e., maize growth stage) which was attributed to the rapid adaptation of soil microbes, such as “Foxy-2”, after repeated exposure to specific agro-ecological conditions (Griffiths and Philippot, 2013).

### 4.2 “Foxy-2” had only a transient suppressive effect on indigenous fungal abundance

“Foxy-2” induced only a transient suppressive effect on abundance of total indigenous fungi in the studied crop rhizospheres, while their community structure remained unaffected. According to Griffiths and Philippot (2013), our results suggested a strong tolerance and resilience potential of the native fungal community towards invading microorganisms (i.e., “Foxy-2”). Similarly, Edel-Hermann et al. (2009) and Savazzini et al. (2009) observed only transient community shifts in indigenous microbial populations in response to inoculation with fungal BCAs (i.e., *Fusarium oxysporum*, *Trichoderma atroviride).*

The suggested suppressive effect of “Foxy-2” on indigenous fungal abundance was superimposed by organic resource availability, but also TC and pH constituting main drivers of total rhizospheric fungal abundance assayed in this study as corroborated by Liu et al. (2015) and Rousk et al. (2009). Furthermore, fungal community structure was mainly determined by maize growth stage, while “Foxy-2” presence did not induce any effect. This finding was verified by Cavaglieri et al. (2009) and Chiarini et al. (1998) confirming the impact of plant development stage on rhizosphere microbial community structures.

### 4.3 Nitrogen-rich organic residues compensate suppressive effects of “Foxy-2”

Interestingly, organic amendments with *T. diversifolia* compensated the transient suppressive effect of “Foxy-2” on indigenous rhizosphere fungal abundance. This implied a competitive situation for resources between “Foxy-2” and indigenous rhizosphere fungi which was supposedly equalized by the provision and accessibility of additional organic N resources (i.e., *T. diversifolia*) promoting the abundance of “Foxy-2” and total fungal community. This interpretation corresponded to previous findings confirming that higher N availability in organic residues (i.e., *T. diversifolia* with low a C/N ratio (Chivenge et al., 2009)) increased total soil fungal abundance in contrast to organic residues with low N availability (high C/N ratio) or mineral fertilizers (España et al., 2011, Kamolmanit et al., 2013, Lee et al., 2013; Zimmermann et al., in press).

## 5 Conclusions

An important intervention of prospective rhizosphere engineering is the use of plant-beneficial microbial inoculants to improve crop yield and health (Dessaux et al., 2016, Quiza et al., 2015, Zhang et al., 2015). The consistent efficacy and environmental safety of these microbial inoculants need to be thoroughly assessed prior to their large scale implementation in contrasting agro-ecosystems (Quiza et al., 2015). The present study successfully identified favoured environmental growth conditions of the rhizosphere-acting BCA “Foxy-2” which will contribute to its proliferation in soils increasing its potential to act effectively against the parasite *S. hermonthica*. Based on our results, persistence and establishment of “Foxy-2” in crop rhizospheres could be appraised if considering site-specific factors such as soil texture, soil carbon background, soil pH and climatic conditions (e.g., rainfall and temperature patterns).

Site-specific conditions could be adjusted in favour of the BCA with increased availability of additional organic N materials if soil resource limitation is prevalent. Moreover, our results indicated that N-rich residues are applicable to compensate a possible resource competition between the BCA and indigenous rhizosphere microorganisms. However, the observed resource competition in this study was only of transient nature and indigenous rhizosphere fungal communities exhibited a strong resilience against “Foxy-2” exposure substantiating the environmental safety of the BCA.

Nonetheless, it needs to be emphasized that measured total fungal abundance was not linked directly with soil fungal activity and functionality (Brankatschk et al., 2011). Hence, prospective studies should focus on the impact of “Foxy-2” on rhizosphere functions mediated by microorganisms (i.e., organic matter decomposition, nutrient cycling) (Musyoki et al., 2015) or specific microbial groups known for their beneficial functions in rhizospheres (e.g., arbuscular mycorrhizal fungi). Moreover, hypothesized biotic factors contributing to soil suppressiveness towards “Foxy-2” such as archaeal and arbuscular mycorrhizal communities need further investigation to decipher the underlying mechanisms of this microbial interaction in crop rhizospheres. We further recommend studying the effect of distinct *S. hermonthica* infestation levels on “Foxy-2” abundance which was neglected in the present study.

## Figures and Tables

**Table 1:** Effects of factors “Field Site”, “Season”, “Treatment” and “Growth stage” and their interactions on gene copy numbers (“Foxy-2”, Total fungi) obtained from qPCR analysis and soil chemical properties. Significant values at *P*<0.05 are highlighted in bold.

		**Factor**	**“Foxy-2” [gene copies g**^**−1**^ **dry soil]**	**Total fungi [gene copies g**^**−1**^**dry soil]**	**TC [g kg**^**−1**^**]**	**N**_**t**_**[g kg**^**−1**^**]**	**EOC [mg kg**^**−1**^**]**	**EON [mg kg**^**−1**^**]**	**NH**_**4+**_**[mg kg**^**−1**^**]**	**NO**_**3−**_**[mg kg**^**−1**^**]**	pH
		Field Site	**0.000**	**0.000**	**0.000**	**0.000**	0.647	0.599	**0.003**	**0.009**	**0.000**
											
Busia		Season (SS)	**0.011**	0.062	**0.000**	**0.000**	0.540	**0.001**	0.081	0.731	**0.000**
	Season 1	Growth stage (EC)	**0.001**	0.142	**0.000**	**0.000**	0.068	0.190	**0.004**	0.119	**0.000**
		Treatment F (F)	–	**0.000**	0.154	0.444	0.746	0.176	0.387	0.644	0.288
		Treatment F+T (FT)	**0.000**	**0.000**	0.214	**0.020**	0.080	0.201	0.723	0.336	**0.000**
		EC×F	–	0.088	**0.006**	0.835	0.977	0.523	0.446	0.992	**0.010**
		EC×FT	**0.000**	0.161	**0.001**	**0.025**	0.062	0.183	0.695	0.624	**0.000**
	Season 2	Growth stage (EC)	0.230	0.285	**0.000**	**0.006**	**0.002**	**0.008**	**0.008**	0.787	**0.000**
		Treatment F (F)	–	0.072	**0.000**	0.061	0.421	0.076	**0.046**	**0.023**	**0.001**
		Treatment F+T (FT)	**0.000**	**0.000**	**0.009**	0.410	0.549	0.980	0.217	0.521	**0.001**
		EC×F	–	0.118	**0.001**	**0.017**	**0.005**	0.145	0.299	**0.049**	**0.000**
		EC×FT	0.238	0.325	**0.038**	0.292	**0.048**	0.849	0.631	0.296	**0.000**
											
Homa Bay		Season (SS)	**0.020**	0.630	**0.001**	**0.000**	0.787	0.550	0.111	**0.003**	**0.000**
	Season 1	Growth stage (EC)	**0.000**	0.093	0.792	**0.001**	0.523	**0.002**	**0.008**	**0.001**	**0.000**
		Treatment F (F)	–	**0.000**	0.344	0.815	0.523	0.331	0.874	0.212	**0.000**
		Treatment F+T (FT)	0.091	**0.000**	0.537	0.078	0.208	0.072	0.663	**0.003**	**0.000**
		EC×F	–	0.096	0.802	0.954	0.406	0.974	0.893	0.073	**0.000**
		EC×FT	0.107	0.130	0.071	**0.011**	0.951	0.176	0.096	0.341	**0.000**
	Season 2	Growth stage (EC)	0.057	0.149	**0.000**	**0.000**	**0.001**	**0.002**	**0.000**	**0.003**	**0.000**
		Treatment F (F)	–	0.084	0.634	**0.022**	**0.037**	0.730	0.406	0.076	**0.000**
		Treatment F+T (FT)	0.120	**0.000**	**0.002**	**0.000**	0.943	0.217	0.601	0.086	**0.000**
		EC×F	–	0.204	0.661	**0.004**	**0.049**	0.464	0.086	0.372	**0.000**
		EC×FT	0.244	0.189	**0.019**	**0.000**	0.597	0.400	0.518	0.297	**0.000**

Abbreviations: F=“Foxy-2”; F+T = “Foxy-2”+*T. diversifolia*. TC: Total carbon, N_t_: Total nitrogen, EOC: Extractable organic carbon,

EON: Extractable organic nitrogen, NH_4_^+^: ammonia, NO_3_^–^: nitrate, pH: soil pH.

**Table 2:** Impact of soil chemical properties on “Foxy-2” and 18S rDNA (Total fungi) gene copy numbers obtained from qPCR analysis. Predicted values calculated by the linear mixed effect model as described in section 2.7 of the manuscript are given in gene copy numbers gram^−1^ dry soil. For every increase of soil chemical property by 1 unit the respective gene copy numbers increase or decrease by the given predicted value in this table. Significant values at *P*<0.05 are highlighted in bold.

**Soil**	**Target gene**	**TC [g kg**^**−1**^**]**	**N**_**t**_**[g kg**^**−1**^**]**	**EOC [mg kg**^**−1**^**]**	**EON [mg kg**^**−1**^**]**	**NH**_**4**_^**+**^**[mg kg**^**−1**^**]**	**NO**_**3**_^**−**^**[mg kg**^**−1**^**]**	**Soil** pH
Across field sites	“Foxy-2”	−770.5 ^ns^	−5.4⁎10^3 ns^	101.2^ns^	−60.9^ns^	**−4.5**⁎**10**^**3**^^ ⁎^	232.4^ns^	**−2.1**⁎**10**^**5 **^^⁎⁎^
	Total fungi	**−4.7**⁎**10**^**7**^	−1.1⁎10^5 ns^	4.8⁎10^4ns^	−7.0⁎10^3 ns^	−8.4⁎10^3 ns^	1.210^4 ns^	**−1.4**⁎**10**^**8**^^ ⁎⁎^
								
Busia	“Foxy-2”	−556.2^ns^	−1.6⁎10^4 ns^	203.5^ns^	**1.8**⁎**10**^**4 **^^⁎^	−138.1^ns^	212.9^ns^	**−1.9**⁎**10**^**4 **^^⁎⁎^
	Total fungi	−7.3⁎10^5 ns^	**5.3**⁎**10**^**7**^	**6.8**⁎**10**^**6**^	−6.4⁎10^4 ns^	−9.1⁎10^3 ns^	2.610^4 ns^	**−4.1**⁎**10**^**8 **^^⁎⁎^
								
Homa Bay	“Foxy-2”	**−3.1**⁎**10**^**3 **^^⁎^	−1.6⁎10^3 ns^	**−7.1**⁎**10**^**3**^^ ⁎^	145.6^ns^	−289.1^ns^	398.7^ns^	**−8.9**⁎**10**^**5**^^ ⁎⁎^
	Total fungi	**−3.8**⁎**10**^**7**^^ ⁎^	2.1⁎10^5 ns^	4.9⁎10^4 ns^	−8.6⁎10^3 ns^	−1.110^4 ns^	8.1⁎10^3 ns^	**−2.5**⁎**10**^**8 **^^⁎⁎^

Significance levels: ns: *P*>0.05; ^⁎^*P*<0.05; ^⁎⁎^*P*<0.01; ^⁎⁎⁎^*P*<0.001.

Abbreviations: “Foxy-2”: “Foxy-2” gene copy numbers; Total Fungi: 18S rDNA gene copy numbers; TC: Total carbon, N_t_: Total ni_t_rogen, EOC: Extractable organic carbon,

EON: Extractable organic nitrogen, NH_4_^+^: ammonia, NO_3_^−^: nitrate.

**Table 3:** Analysis of similarity (ANOSIM) of total fungal TRFLP datasets based on global *R* values for the factors treatment and maize growth stage and single *R* values for pair wise comparison within treatments and maize growth stages. The magnitude of *R* indicates the degree of separation between two tested communities. An *R* score of 1 indicates a complete separation, while 0 indicates no separation.

Soil	Factor	Global *R*

Busia vs. Homa Bay	–	1.0^⁎⁎⁎^
Busia	Treatment	0.073^ns^
	Maize growth stage	0.627^⁎⁎^
Homa Bay	Treatment	0.119^ns^
	Maize growth stage	0.410

Soil	Treatment	*R* statistic
	(pair wise comparison)	

Busia	C+S vs. F+S	0.074^ns^
	C+S vs. F+S+T	0.160^ns^
	F+S vs. F+S+T	0.173^ns^
Homa Bay	C+S vs. F+S	0.086^ns^
	C+S vs. F+S+T	0.049^ns^
	F+S vs. F+S+T	0.184^ns^

Soil	Maize growth stage	*R* statistic
	(pair wise comparison)	

Busia	EC30 vs. EC60	0.753^⁎⁎^
	EC30 vs. EC90	0.840^⁎⁎^
	EC60 vs. EC90	0.420^⁎^
Homa Bay	EC30 vs. EC60	0.395^⁎^
	EC30 vs. EC90	0.333^⁎^
	EC60 vs. EC90	0.580^⁎⁎^

Significance levels: ns: *P*>0.05; ^⁎^*P*<0.05; ^⁎⁎^*P*<0.01; ^⁎⁎⁎^*P*<0.001.

Treatment codes: C+S=uncoated maize+*S. hermonthica*, F+S=coated maize (with “Foxy-2”)+*S. hermonthica* and F+S+T=coated maize+*S. hermonthica*+*T. diversofolia.* EC30=early leaf development stage, EC60=flowering stage, EC90=senescence stage.

## References

[bib1] Abbasher A.A., Kroschel J., Sauerborn J. (1995). Microorganisms of *Striga hermonthica* in Northern Ghana with Potential as Biocontrol Agents. Biocontrol Sci. Technol..

[bib500] Anderson M.J., Willis T.J. (2003). Canonical analysis of principal coordinates: a useful method of constrained ordination for ecology. Ecology.

[bib2] Avedi E.K., Ochieno D.M.W., Ajanga S., Wanyama C., Wainwright H., Elzein A., Beed F. (2014). Fusarium oxysporum f. sp. strigae strain Foxy 2 did not achieve biological control of *Striga hermonthica* parasitizing maize in Western Kenya. Biol. Control.

[bib3] Bengtson P., Sterngren A.E., Rousk J. (2012). Archaeal abundance across a pH gradient in an Arable Soil and its relationship to bacterial and fungal growth rates. Appl. Environ. Microbiol..

[bib4] Brankatschk R., Töwe S., Kleineidam K., Schloter M., Zeyer J. (2011). Abundances and potential activities of nitrogen cycling microbial communities along a chronosequence of a glacier forefield. ISME J..

[bib5] Cavaglieri L., Orlando J., Etcheverry M. (2009). Rhizosphere microbial community structure at different maize plant growth stages and root locations. Microbiol. Res..

[bib6] Chiarini L., Bevivino A., Dalmastri C., Nacamulli C., Tabacchioni S. (1998). Influence of plant development, cultivar and soil type on microbial colonization of maize roots. Appl. Soil Ecol..

[bib7] Chivenge P., Vanlauwe B., Gentile R., Wangechi H., Mugendi D., Kessel C., van, Six J. (2009). Organic and mineral input management to enhance crop productivity in central Kenya. Agron. J..

[bib8] Clarke K.R., Gorley R.N. (2006). PRIMER v6: User Manual/Tutorial.

[bib9] Compant S., Duffy B., Nowak J., Clément C., Barka E.A. (2005). Use of plant growth-promoting bacteria for biocontrol of plant diseases: principles, mechanisms of action, and future prospects. Appl. Environ. Microbiol..

[bib10] de Groote H., Wangare L., Kanampiu F., Odendo M., Diallo A., Karaya H., Friesen D. (2008). The potential of a herbicide resistant maize technology for Striga control in Africa. Agric. Syst..

[bib11] Dessaux Y., Grandclément C., Faure D. (2016). Engineering the Rhizosphere.

[bib12] Dunbar J., Ticknor L.O., Kuske C.R. (2000). Assessment of microbial diversity in four Southwestern United States soils by 16S rRNA gene terminal restriction fragment Analysis. Appl. Environ. Microbiol..

[bib13] Edel-Hermann V., Brenot S., Gautheron N., Aimé S., Alabouvette C., Steinberg C. (2009). Ecological fitness of the biocontrol agent *Fusarium oxysporum* Fo47 in soil and its impact on the soil microbial communities. FEMS Microbiol. Ecol..

[bib501] Ejeta G. (2007). The Striga scourge in Africa: a growing pandemic. in: Integrating New Technologies for Striga Control: Towards Ending the Witch-Hunt.

[bib14] Elzein A., Kroschel J. (2004). Fusarium oxysporum Foxy 2 shows potential to control both *Striga hermonthica* and *S. asiatica*. Weed Res..

[bib15] España M., Rasche F., Kandeler E., Brune T., Rodriguez B., Bending G.D., Cadisch G. (2011). Assessing the effect of organic residue quality on active decomposing fungi in a tropical Vertisol using 15N-DNA stable isotope probing. Fungal Ecol..

[bib16] Fang X., You M.P., Barbetti M.J. (2012). Reduced severity and impact of Fusarium wilt on strawberry by manipulation of soil pH, soil organic amendments and crop rotation. Eur. J. Plant Pathol..

[bib17] FAO), 2006. Guidelines for the export, shipment, import and release of biological control agents and other beneficial organisms (ISPM No. 3). In: International Standards for Phytosanitary Measures. FAO, Rome.

[bib18] Farooq U., Bano A. (2013). Screening of indigenous bacteria from rhizosphere of maize (Zea mays L.) for their plant growth promotion ability and antagonism against fungal and bacterial pathogens. J. Anim. Plant Sci..

[bib19] Fredriksson N.J., Hermansson M., Wilén B.-M. (2014). Impact of T-RFLP data analysis choices on assessments of microbial community structure and dynamics. BMC. Bioinformatics.

[bib20] Gacheru E., Rao M.R. (2001). Managing Striga infestation on maize using organic and inorganic nutrient sources in western Kenya. Int. J. Pest Manag..

[bib21] Gerbore J., Benhamou N., Vallance J., Floch G.L., Grizard D., Regnault-Roger C., Rey P. (2013). Biological control of plant pathogens: advantages and limitations seen through the case study of Pythium oligandrum. Environ. Sci. Pollut. Res..

[bib22] Griffiths B.S., Philippot L. (2013). Insights into the resistance and resilience of the soil microbial community. FEMS Microbiol. Rev..

[bib23] Grigera M., Drijber R.A., Wienhold B.J. (2007). Increased abundance of arbuscular mycorrhizal fungi in soil coincides with the reproductive stages of maize. Soil Biol. Biochem..

[bib24] Hai B., Diallo N.H., Sall S., Haesler F., Schauss K., Bonzi M., Assigbetse K., Chotte J.-L., Munch J.C., Schloter M. (2009). Quantification of Key genes steering the microbial nitrogen cycle in the rhizosphere of sorghum cultivars in tropical agroecosystems. Appl. Environ. Microbiol..

[bib25] Höper H., Steinberg C., Alabouvette C. (1995). Involvement of clay type and pH in the mechanisms of soil suppressiveness to fusarium wilt of flax. Soil Biol. Biochem..

[bib26] Hu J.-L., Lin X.-G., Wang J.-H., Shen W.-S., Wu S., Peng S.-P., Mao T.-T. (2010). Arbuscular Mycorrhizal fungal inoculation enhances suppression of Cucumber Fusarium Wilt in greenhouse soils. Pedosphere.

[bib27] IUSS Working Group WRB (2015). World Reference Base for Soil Resources 2014, Update 2015 International Soil Classification System for naming Soils and creating Legends for Soil Maps.

[bib28] Kamolmanit B., Vityakon P., Kaewpradit W., Cadisch G., Rasche F. (2013). Soil fungal communities and enzyme activities in a sandy, highly weathered tropical soil treated with biochemically contrasting organic inputs. Biol. Fertil. Soils.

[bib29] Kanampiu F.K., Ransom J.K., Friesen D., Gressel J. (2002). Imazapyr and pyrithiobac movement in soil and from maize seed coats to control Striga in legume intercropping. Crop Prot..

[bib30] Köhl J., Lombaers C., Moretti A., Bandyopadhyay R., Somma S., Kastelein P. (2015). Analysis of microbial taxonomical groups present in maize stalks suppressive to colonization by toxigenic Fusarium spp.: a strategy for the identification of potential antagonists. Biol. Control.

[bib31] Lee Y.H., Kim M.K., Lee J., Heo J.Y., Kang T.H., Kim H., Yun H.D. (2013). Organic fertilizer application increases biomass and proportion of fungi in the soil microbial community in a minimum tillage Chinese cabbage field. Can. J. Soil Sci..

[bib32] Legendre P., Anderson M.J. (1999). Distance-based redundancy analysis: testing multispecies responses in multifactorial ecological experiments. Ecol. Monogr..

[bib33] Liu J., Maldonado-Mendoza I., Lopez-Meyer M., Cheung F., Town C.D., Harrison M.J. (2007). Arbuscular mycorrhizal symbiosis is accompanied by local and systemic alterations in gene expression and an increase in disease resistance in the shoots. Plant J..

[bib34] Liu J., Sui Y., Yu Z., Shi Y., Chu H., Jin J., Liu X., Wang G. (2015). Soil carbon content drives the biogeographical distribution of fungal communities in the black soil zone of northeast China. Soil Biol. Biochem..

[bib35] Marley P.S., Aba D.A., Shebayan J. a Y., Musa R., Sanni A. (2004). Integrated management of Striga hermonthica in sorghum using a mycoherbicide and host plant resistance in the Nigerian Sudano-Sahelian savanna. Weed Res..

[bib36] Marschner P., Kandeler E., Marschner B. (2003). Structure and function of the soil microbial community in a long-term fertilizer experiment. Soil Biol. Biochem..

[bib37] Milling A., Smalla K., Maidl F.X., Schloter M., Munch J.C. (2005). Effects of transgenic potatoes with an altered starch composition on the diversity of soil and rhizosphere bacteria and fungi. Plant Soil.

[bib38] Musyoki M.K., Cadisch G., Enowashu E., Zimmermann J., Muema E., Beed F., Rasche F. (2015). Promoting effect of Fusarium oxysporum [f.sp. strigae] on abundance of nitrifying prokaryotes in a maize rhizosphere across soil types. Biol. Control.

[bib39] Musyoki M.K., Cadisch G., Zimmermann J., Wainwright H., Beed F., Rasche F. (2016). Soil properties, seasonality and crop growth stage exert a stronger effect on rhizosphere prokaryotes than the fungal biocontrol agent *Fusarium oxysporum* f.sp. strigae. Appl. Soil Ecol..

[bib40] Ndambi B., Cadisch G., Elzein A., Heller A. (2011). Colonization and control of Striga hermonthica by Fusarium oxysporum f. sp. strigae, a mycoherbicide component: an anatomical study. Biol. Control.

[bib41] OECD (2014). OECD Guidance to the Environmental Safety Evaluation of Microbial Biocontrol Agents, Series on Pesticides and Biocides.

[bib42] Pereira e Silva M.C., Dias A.C.F., van Elsas, Salles J.F. (2012). Spatial and temporal variation of archaeal, bacterial and fungal communities in agricultural soils. PLoS One.

[bib502] Pinheiro J., Bates D., DebRoy S., Sarkar D., R Core Team (2016). nlme: Linear and Nonlinear Mixed Effects Models. R package version.

[bib43] Quiza L., St-Arnaud M., Yergeau E. (2015). Harnessing phytomicrobiome signaling for rhizosphere microbiome engineering. Front. Plant Sci..

[bib44] Rasche F., Hödl V., Poll C., Kandeler E., Gerzabek M.H., Elsas J.D., van, Sessitsch A. (2006). Rhizosphere bacteria affected by transgenic potatoes with antibacterial activities compared with the effects of soil, wild-type potatoes, vegetation stage and pathogen exposure. FEMS Microbiol. Ecol..

[bib45] Rasche F., Musyoki M.K., Röhl C., Muema E.K., Vanlauwe B., Cadisch G. (2014). Lasting influence of biochemically contrasting organic inputs on abundance and community structure of total and proteolytic bacteria in tropical soils. Soil Biol. Biochem..

[bib46] Rees G.N., Baldwin D.S., Watson G.O., Perryman S., Nielsen D.L. (2005). Ordination and significance testing of microbial community composition derived from terminal restriction fragment length polymorphisms: application of multivariate statistics. Antonie Van Leeuwenhoek.

[bib47] Rousk J., Brookes P.C., Bååth E. (2009). Contrasting Soil pH Effects on Fungal and Bacterial Growth Suggest Functional Redundancy in Carbon Mineralization. Appl. Environ. Microbiol..

[bib48] Savazzini F., Longa C.M.O., Pertot I. (2009). Impact of the biocontrol agent Trichoderma atroviride SC1 on soil microbial communities of a vineyard in northern Italy. Soil Biol. Biochem..

[bib49] Schaub B., Marley P., Elzein A., Kroschel J. (2006). Field evaluation of an integrated *Striga hermontica* management in Sub-Saharan Africa: synergy between Striga-mycoherbicides (biocontrol) and sorghum and maize resistant varieties. J. Plant Dis. Prot..

[bib50] Senechkin I.V., van Overbeek L.S., van Bruggen A.H.C. (2014). Greater Fusarium wilt suppression after complex than after simple organic amendments as affected by soil pH, total carbon and ammonia-oxidizing bacteria. Appl. Soil Ecol..

[bib51] Shukla A., Dehariya K., Vyas D., Jha A. (2015). Interactions between arbuscular mycorrhizae and *Fusarium oxysporum* f. sp. ciceris: effects on fungal development, seedling growth and wilt disease suppression in *Cicer arietinum* L. Arch. Phytopathol. Plant Prot..

[bib52] Vainio E.J., Hantula J. (2000). Direct analysis of wood-inhabiting fungi using denaturing gradient gel electrophoresis of amplified ribosomal DNA. Mycol. Res..

[bib53] van der Heijden M.G.A., Bardgett R.D., Van Straalen N.M. (2008). The unseen majority: soil microbes as drivers of plant diversity and productivity in terrestrial ecosystems. Ecol. Lett..

[bib54] Vanlauwe B., Kanampiu F., Odhiambo G.D., De Groote H., Wadhams L.J., Khan Z.R. (2008). Integrated management of *Striga hermonthica*, stemborers, and declining soil fertility in western Kenya. Field Crops Res.

[bib55] Velivelli S.L.S., De Vos P., Kromann P., Declerck S., Prestwich B.D. (2014). Biological control agents: from field to market, problems, and challenges. Trends Biotechnol..

[bib504] Venables W.N., Ripley B.D. (2002). Modern Applied Statistics with S, Statistics and Computing.

[bib56] Venne J., Beed F., Avocanh A., Watson A. (2009). Integrating *Fusarium oxysporum* f. sp. strigae into cereal cropping systems in Africa. Pest Manag. Sci..

[bib57] Yergeau E., Labour K., Hamel C., Vujanovic V., Nakano-Hylander A., Jeannotte R., St-Arnaud M. (2010). Patterns of Fusarium community structure and abundance in relation to spatial, abiotic and biotic factors in soil. FEMS Microbiol. Ecol..

[bib58] Zadoks J.C., Chang T.T., Konzak C.F. (1974). A decimal code for the growth stages of cereals. Weed Res..

[bib59] Zhang Y., Ruyter-Spira C., Bouwmeester H.J. (2015). Engineering the plant rhizosphere. Curr. Opin. Biotechnol..

[bib60] Zimmermann J., de Klerk M., Musyoki M.K., Viljoen A., Watson A.K., Beed F., Gorfer M., Cadisch G., Rasche F. (2015). An explicit AFLP-based marker for monitoring *Fusarium oxysporum* f.sp. strigae in tropical soils. Biol. Control.

